# The *Why We Retweet* scale

**DOI:** 10.1371/journal.pone.0206076

**Published:** 2018-10-18

**Authors:** Anuja Majmundar, Jon-Patrick Allem, Tess Boley Cruz, Jennifer Beth Unger

**Affiliations:** Keck School of Medicine of USC, Los Angeles, CA, United States; Dalian University of Technology, CHINA

## Abstract

**Background:**

Twitter offers a platform for rapid diffusion of information and its users' attitudes and behaviors. Insights about information propagation via retweets (the message forwarding function) offer observable explanations of ways in which modern human interactions get organized in the form of online networks, and contextualized in the form of public health, policy decisions, disaster management, and civic participation. This study conceptualized and validated the Why We Retweet Scale to contextualize retweeting behavior.

**Objective:**

Twitter users were identified using clustering algorithms that consider a users’ position in their network and invited for an online survey. Participants (N = 1433) responded to 19 questions about why they retweet. Exploratory factor Analysis (EFA) was conducted on a scale development sample (70% of original sample), which informed the Confirmatory Factor Analysis (CFA) on a scale testing sample (30% of the original sample). Varimax rotation was used to obtain a rotated factor solution, which resulted in interpretable factors. Demographic differences among scale factors were analyzed using one-way ANOVA or independent samples t-tests.

**Results:**

The final model (χ^2^_21_ = 28, RMSEA = .03 [90% CI, 0.00–0.06], CFA = .99, TLI = 0.99) represented a parsimonious solution with 4 factors, measured by 2–3 items each, creating a final scale consisting of 9 items. Factor labels and definitions were: (1) *Show approval*, “Show support to the tweeter”; (2) *Argue*, “To argue against a tweet that I disagree with”; (3) *Gain attention*, “Add followers or gain attention”; and (4) *Entertain*, “Create humor/amusement”. Demographic differences were also reported.

**Conclusions:**

The *Why We Retweet Scale* offers a useful conceptualization and assessment of motivations for retweeting. In the future, communication strategists might consider the factors associated with information propagation when designing campaign messages to maximize message reach and engagement on Twitter.

## Introduction

Social media platforms leverage the power of online networks for information seeking and sharing[1–7]. Twitter–a micro blogging service, offers opportunities to “tweet” by authoring original content or “retweet” by reposting another user’s tweet. Retweeting is a particularly powerful tool for widespread diffusion of information[8–10]. An analysis of retweets offers insights about how users interact with one another and with diverse types of information. From an information dissemination perspective, retweeters have been described as either information creators (professional or non-professional mass media accounts related to a popular event), promoters (superstars), supporters (average users with some influence to stimulate discussion among friends) and/or consumers (users who retweet more than tweet original content)[11]. These insights about users and information propagation via retweets offer observable explanations of ways in which modern human interactions get organized in the form of online networks, and contextualized in the form of public health, e.g., bird flu information[12], and diffusion of e-cigarette marketing messages[13], policy decisions, e.g., the recent net neutrality debate[14], disaster management, e.g., hurricanes/typhoons[15], environment, e.g. climate change[16], and civic participation, e.g., local tobacco regulations[17].

Despite the importance of retweeting for information diffusion and ways in which online communication is organized, limited research exists on why people retweet. In a recent study, Boyd et al. summarize ten different retweeting motivations (e.g., To spread tweets to new audiences, to validate others’ thoughts, to publicly agree with someone) [18]. These categories of motivation for retweeting were based on a qualitative analysis of responses to a question, “What do you think are the different reasons for why people RT something”, from one author’s twitter account. These categories offered useful direction for the development of an updated and in-depth inquiry that incorporates perspectives of actual Twitter users. Earlier studies suggest that retweeting is driven more by interpersonal, rather than for topic- or interaction-oriented, ends [19]. Retweeting is also explained using social cognitive theory to examine information sharing self-efficacy, attachment motivation and critical mass as its antecedents [20]; and using social communication theory, to highlight social tie strength and topical relevance with the message receiver as the most influential factors driving retweeting [21].

Recent work has also applied retweeting motivations in specific contexts. For example, during disaster situations, users usually retweet to share information, to convey the significance of information in their network, to express their feelings, or to get feedback/alert other people[22]. A conceptualization and assessment of retweeting motivations can offer valuable extensions to the current literature. In the present study, we developed the *Why We Retweet Scale* to measure individual perceptions about what drives Twitter users to retweet. This study’s goals were to (a) construct the *Why We Retweet Scale*, (b) determine the psychometric properties of this scale, and (c) determine whether reasons for retweeting vary across demographic subgroups. Ultimately, a validated scale will inform future investigations related to retweeting motivations in the context of decision making for communication campaigns.

## Methods

### Data collection

Surveys were completed by (n = 1433) participants from a study focused on the health behaviors of Twitter users who discuss tobacco-related products (e.g., e-cigarettes, cigarettes, etc.). Initially, Twitter posts were obtained through Twitter’s Streaming API. Along with the text of the tweet, this data included the username of the person who posted the tweet and whether the tweet was an original tweet or a retweet. Each retweet was labeled with the username who retweeted the message and the user who originally posted the message. To create a diverse stratified sample, this information was used to construct the social network structure of users, where connections between users were defined by retweets of messages from one user to another. From this retweet network, clusters were identified by conducting a modularity analysis, which helped locate clusters within a network by grouping nodes (i.e., Twitter users) who have more connections (i.e., retweets) with others within a group than those outside of the group. From each cluster, *Opinion leaders* were chosen as those who had been retweeted the most; *Followers* were identified within each cluster as those who had retweeted others the most. *Random users* were independently found by Twitter’s API get-user-status function, which returns users who have recently posted a tweet, from which a sample was randomly selected. The goal of this procedure was to make sure we included a variety of Twitter users based on their positions in the Twitter network; 24.2% of the participants were categorized as *Opinion Leaders*, 39.6% as *Followers*, and 36.2% as *Random users* in the retweet network

From January-December 2016, Twitter users identified in the above networks were sent private messages inviting them to participate in a survey on health behaviors and reasons for retweeting among other survey items. After consenting to participate, each participant was directed to the online survey. All participants were over 18 years, residing in the United States, able to complete an online survey in English, and received a $20 gift card for completing the survey. The University of Southern California Institutional Review Board approved all study procedures. All analyses adhered to the terms and conditions, terms of use, and privacy policies of Twitter.

## Measures

### Scale items

Participants responded to 19 questions that were developed to understand why people retweet. Response options were provided on a scale of 1–5 with “Never” coded 1 and “Very often” coded 4, ‘Prefer not to answer’ coded as missing (Table 1). These items were based on boyd et. al’s categories [18], consultation with social scientists with expertise in social media research, and a focus group of Twitter users.

**Table 1:** *Why We Retweet Scale* items.

No.	Item
1.	To show that I saw the tweet
2.	To make more people see the tweet
3.	To spread knowledge
4.	To entertain
5.	To share a funny joke
6.	To make my own twitter feed look good
7.	To add my thoughts to a tweet
8.	To get my followers to join the discussion
9.	To say that I agree with the tweet
10.	To argue against a tweet that I disagree with
11.	To introduce my followers to the tweeter
12.	To show my support for the tweeter
13.	To show my followers that I like the tweeter
14.	To show my followers how I feel about an issue
15.	To tell my followers about an event
16.	To gain new followers
17.	To get someone's attentions
18.	To save tweets so I can find them again
19.	Because I trust the tweeter

### Demographic measures

Participants were asked to indicate their gender (male, female), age (years), race (White, Black or African American, American Indian or Alaska Native, Asian/Pacific Islander, or Other) and ethnicity (Hispanic/Non-Hispanic), income (Less than $10,000; $10,000 to $14,999; up to $200,000 or more in increments of $10,000 per year), level of education (Less than high school; some high school, no diploma; GED; High school graduate—diploma; Some college but no degree; Associate degree-occupational/vocational; associate degree—academic program; bachelor's degree (ex: BA, AB, BS); master's degree (ex: MA, MS, MEng,Med, MSW); professional school degree (ex: MD, DDS, DVM, JD); Doctorate degree (ex: PhD, EdD)) S1 Table. Those who did not wish to answer selected the option ‘Prefer not to answer’ for all the above questions except sex and age.

### Procedure

All statistical analyses were performed using Stata version 14.2. Responses indicating ‘prefer not to answer’ were marked as missing. Complete cases were randomly drawn to populate the scale development sample (70% of total sample, N = 1003) and scale validation sample (30% of total sample, N = 430). The analytic sample for Exploratory Factor Analysis (EFA) was n = 824 due to listwise deletion to handle nonresponse or missing items, while the analytic sample for Confirmatory Factor Analysis (CFA) was n = 366. First, EFA was performed on the scale development sample to determine the optimal number of factors that could account for the observed variation in responses. Factor correlations less than 0.3 implied that the solution remained orthogonal [23]. We centered all scale items on their means and the Kaiser-Meyer-Olkin measure of sampling adequacy was assessed to determine how well the correlation between pairs of variables was explained by other variables in the analysis [24]. The Bartlett test of sphericity was used to test the null hypothesis that the observed correlation matrix was an identity matrix corresponding to no correlation between scale items. The EFA used principal components analysis. Factors with eigenvalues greater than one were extracted. Items with factor loadings greater than 0.7 were retained as indicators of their respective factors.

Next, to validate the scale, confirmatory factor analysis was conducted on the validation sample. The criteria for model fit were CFI was greater than 0.9 and RMSEA <0.05 [25]. The maximum-likelihood estimation procedure was employed as a global test of the model [26]. Each subscale’s internal reliability was assessed using Cronbach’s alpha and/or Spearman-brown’s coefficient (in the case of two-item factors) [27]. Items that loaded on each factor were summed to create a factor score. Construct validity, in particular, convergent validity was assessed based on the average variance extracted (AVE) for each factor. For factors with AVE less than 0.5, a composite reliability higher than 0.6 was considered adequate for establishing convergent validity [28]. Additionally, squared inter-factor correlations for each factor were compared with the corresponding squared root of AVE scores to establish discriminant validity [29]. Lastly, independent samples t-tests and ANOVA tests were used on the complete analytic sample (N = 1190) to analyze demographic differences on each subscale.

## Results

Participants were predominantly female (54%), White (63.7%), non-Hispanic (76.5%), earned less than $35,000 per year (54%), with a mean age of about 23.8 years (S.D. = 8.8) and about 35% graduated from high school with a diploma.

### Internal consistency and exploratory factor analysis

Bartlett’s test of sphericity yielded a large value (7835.64) and the associated significance probability (p = 0.001) indicated that the observed correlation analyses were statistically significant. Additionally, Kaiser-Meyer-Olkin’s value was 0.87 which justified further analysis. Initial item analysis was performed on all 19 items on a training sample. It was determined that the solution remained orthogonal. Varimax rotation was performed on all 19-items on the same training sample. Principal components analysis was performed and produced a four-factor solution with eigenvalues greater than 1 based on Kaiser’s criteria (cumulative variance explained = 55%). Factor loadings greater than or equal to 0.7 were retained for interpretation. Table 2 reports resulting factor loadings of 19 items.

**Table 2:** Exploratory factor analyses item loadings (Varimax rotation) (N Training = 824).

No.	Item	Factor1 Show approval	Factor2 Argue	Factor 3 Gain attention	Factor 4 Entertain
1.	To show that I saw the tweet				
2.	To make more people see the tweet				
3.	To Spread knowledge				
4.	To entertain				0.88
5.	To share a funny joke				0.90
6.	To make my own twitter feed look good				
7.	To add my thoughts to a tweet		0.70		
8.	To get my followers to join the discussion				
9.	To say that I agree with the tweet				
10.	To argue against a tweet that I disagree with		0.72		
11.	To introduce my followers to the tweeter	0.70			
12.	To show my support for the tweeter	0.77			
13.	To show my followers that I like the tweeter	0.73			
14.	To show my followers how I feel about an issue				
15.	To tell my followers about an event				
16.	To gain new followers			0.74	
17.	To get someone's attentions			0.71	
18.	To save tweets so I can find them again				
19.	Because I trust the tweeter				

Exploratory Factory Analysis resulted in 4 interpretable factors. Colored cells indicate factor loadings ≥ 0.7 for the corresponding items (Column 1).

The rotated factor solution resulted in four interpretable factors. Factor labels and items were: Factor 1 *Show approval*, “To show my support for the tweeter” (explained 24% of the variance); Factor 2 *Argue*, “To argue against a tweet that I disagree with” (explained 22% of the variance); Factor 3 *Gain attention*, “Add followers or gain attention” (explained 14% of the variance); Factor 4 *Entertain*, “To entertain” (explained 14% of the variance).

### Confirmatory factor analysis

To confirm findings from the EFA, a CFA model was fit using 9 items and 4 factors, with each of the items only allowed to load and be freely estimated on its hypothesized factor. The final model (χ^2^_21_ = 28, RMSEA = .03 [90% CI, 0.00–0.06], CFA = .99, TLI = 0.99) represented a parsimonious solution with 4 factors, measured by 2–3 items each, creating a final scale consisting of 9 items [30]. This solution offered a good fit without any adjustments, such as covarying parameters or allowing variables to load on additional factors, to achieve the final model. Individual item loadings were high for all items on their respective factors (range = 0.70–0.93; see Table 3).

**Table 3:** Confirmatory factor analyses item loadings, and Cronbach coefficient alpha for 4 factors. (NValidation = 366).

Item	Factor1 Show approval	Factor2 Argue	Factor 3 Gain attention	Factor 4 Entertain
To entertain				0.93
To share a funny joke				0.77
To add my thoughts to a tweet		0.70		
To argue against a tweet that I disagree with		0.71		
To introduce my followers to the tweeter	0.71			
To show my support for the tweeter	0.73			
To show my followers that I like the tweeter	0.86			
To gain new followers			0.81	
To get someone's attentions			0.83	
**Cronbach’s alpha**	0.81	0.60	0.84	0.80
**Spearman-Brown co-efficient**	-	0.69	0.89	0.86
**Average variance extracted**	0.60	0.42	0.67	0.73

Colored cells indicate factor loadings ≥ 0.7 for the corresponding items (Column 1). Cronbach’s alpha (above) indicate reliability coefficients for each factor in the Confirmatory Factor Analysis. Spearman-Brown co-efficient is reported for two-item factors. Average variance extracted explain the extent to which each factor explains the variance of its indicators.

### Factor inter-correlations and internal consistency

Pearson’s product-moment correlations were assessed for each pair of subfactor scores. All factors were correlated significantly (p < .05) from 0.19 to 0.45. Internal consistency was also acceptable for each factor, as measured by Cronbach’s alpha (range = 0.6 to .084; see Table 3). We examined the Spearman-Brown coefficient for all two-item factors to predict their reliability for a 3-item test (See Table 3). As noted in Table 3, Factor 2 –*Argue* Spearman-Brown co-efficient is 0.69, which is lower but approaches an acceptable coefficient of 0.70.

### Construct validity

The scale’s construct validity was assessed in terms of convergent and discriminant validity. Convergent validity was assessed based on the average variance extracted (AVE) for each factor. AVE values were higher than 0.5 all except one factor, Argue (AVE = 0.42; Table 3). However, the composite reliability (CR) of this factor was 0.59, indicating that it approached an acceptable level of convergent validity (see Table 4). Discriminant validity was determined to be sufficiently high for the scale, given the square root of the AVE values was higher than the inter-factor squared correlations were (see Table 4).

**Table 4:** Composite reliability, square root of factor AVE, and squared correlations between factors.

	Composite Reliability	Square root AVE	Factor1 Show approval	Factor2 Argue	Factor 3 Gain attention	Factor 4 Entertain
**Factor1** **Show approval**	0.81	0.77	1.00			
**Factor2** **Argue**	0.59	0.65	0.28	1.00		
**Factor 3** **Gain attention**	0.85	0.82	0.37	0.33	1.00	
**Factor 4** **Entertain**	0.84	0.85	0.08	0.17	0.07	1.00

Composite Reliability and AVE for each factor assess the convergent validity of a scale.

Squared root AVE for each factor is compared with inter-factor squared correlations to assess discriminant validity.

### Relationship between *Why We Retweet Scale* with demographic characteristics

**Fig 1:**
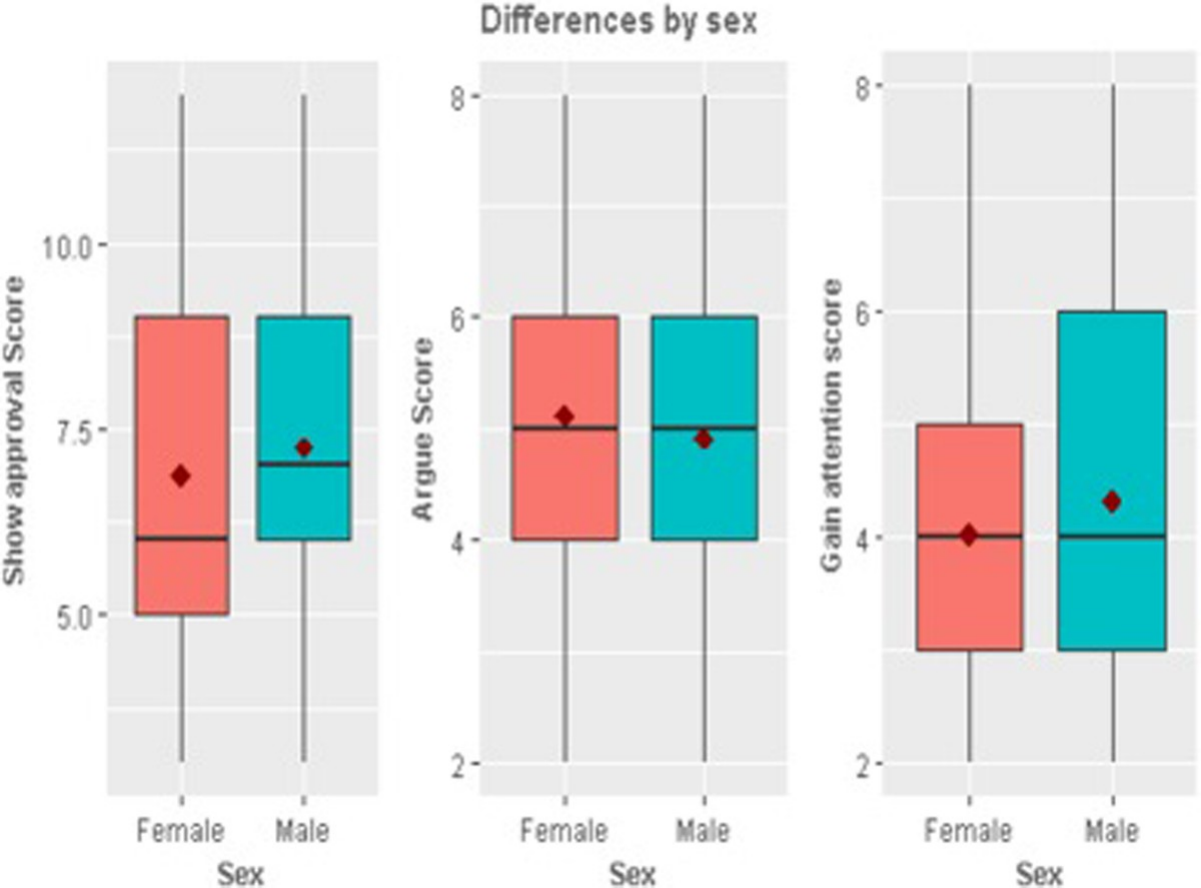
Significant differences by sex.

**Fig 2:**
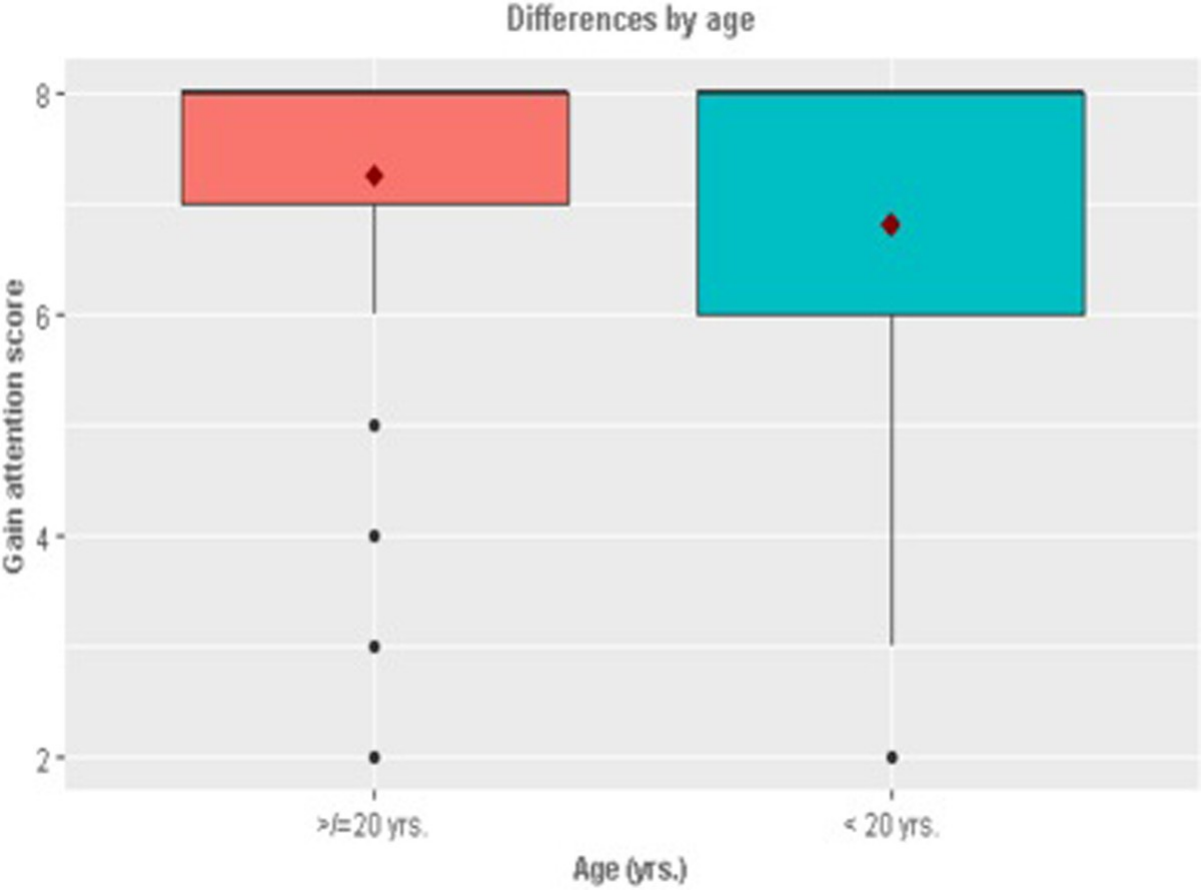
Significant differences by age.

**Fig 3:**
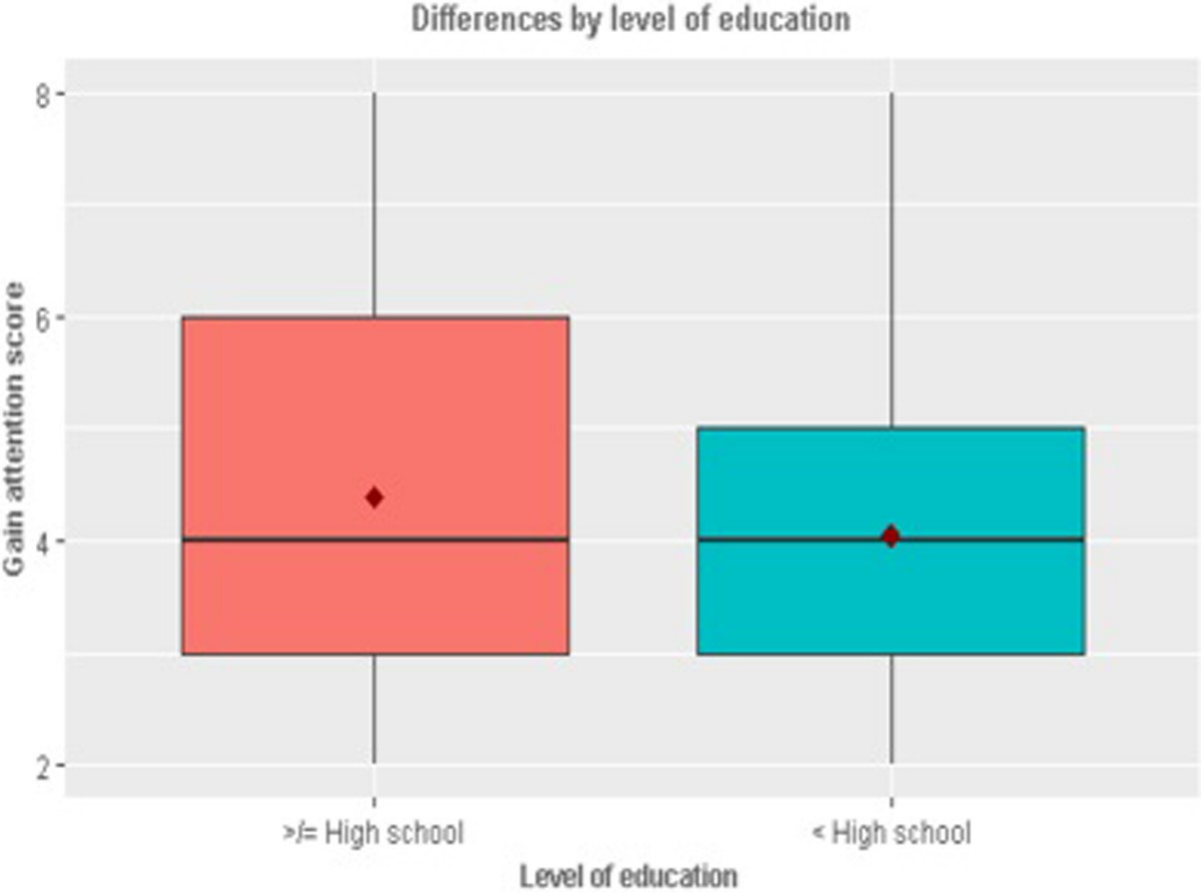
Significant differences by education.

**Fig 4:**
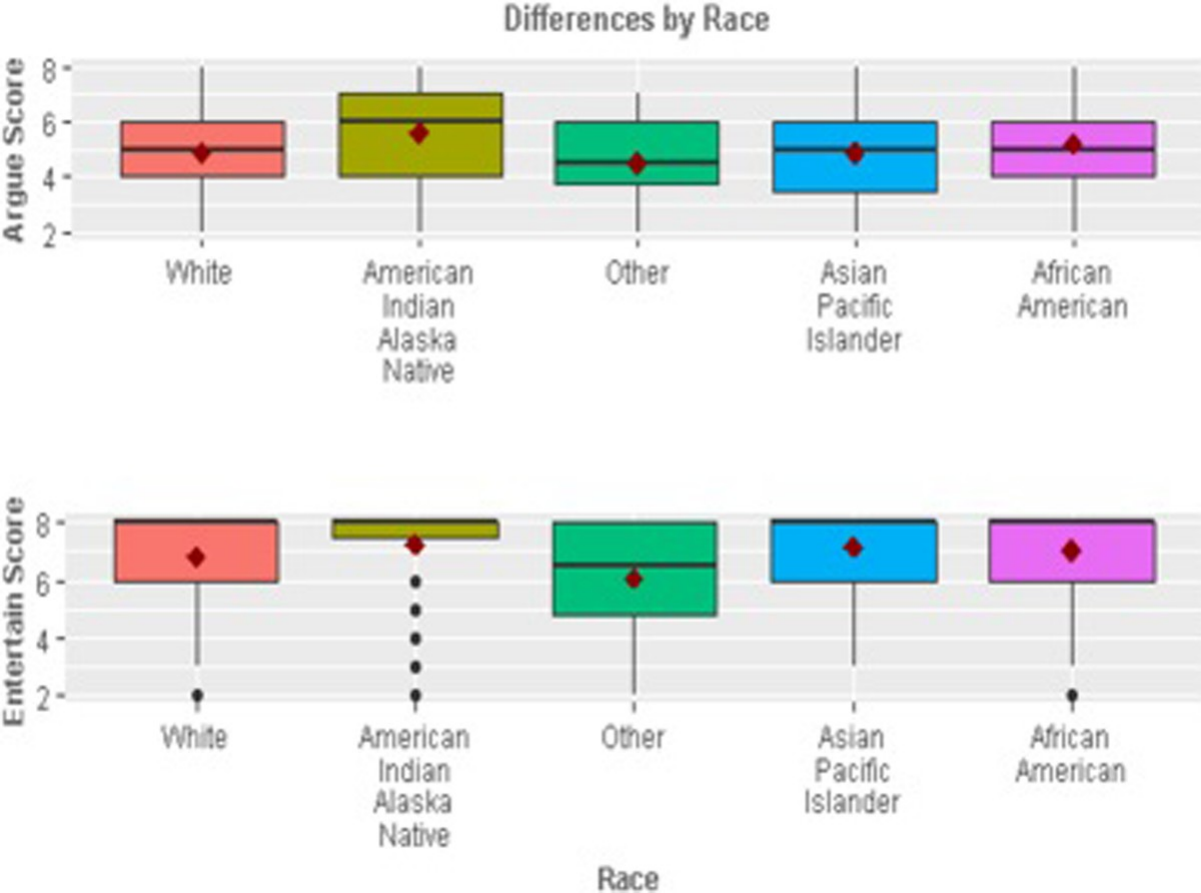
Significant differences by race.

**Fig 5:**
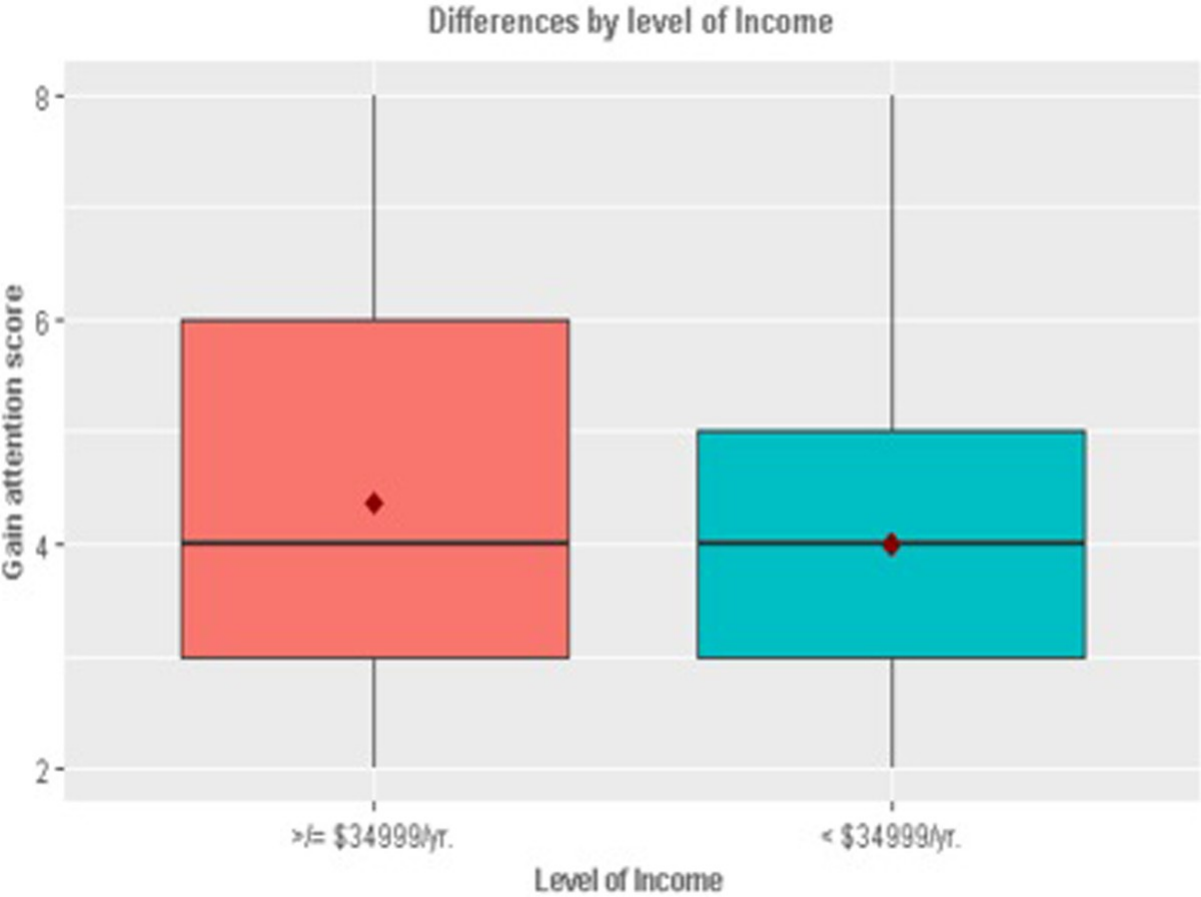
Significant differences by income.

In terms of the demographic differences (Figs 1–5), those who retweeted to *Show approval* (t_924_ = -2.05, p = 0.04) and *Gain attention* (t_924_ = -2.62, p = 0.001) were more likely to be men than women. However, those who retweeted to *Argue* were more likely to be women than men (t_924_ = 2.14, p = 0.03). Those who retweeted to *Argue* (F = 4.99, p = 0.001) *or Entertain* (F = 3.11, p = 0.01) were more likely to be African American than other races (F = 4.99, p = 0.001). Those who retweeted to *Gain attention* were likely to be less educated (t_902_ = 2.58, p = 0.01) and earning a lower annual income of less than $34,999 per year (t_764_ = 2.42, p = 0.01). Those who retweeted to *Entertain* were more likely to be younger (less than or equal to 20 years of age).

## Discussion

The present study conceptualized and validated the *Why We Retweet Scale*, offering insights into the nature and dimensionality of the motivations for retweeting, and provided an empirical investigation of boyd et al.’s exploratory, qualitative study[18]. While boyd et al reported on ten different motivations to retweet, the present study suggested that retweeting is driven by four factors: *Show approval*, *Argue*, *Gain Attention and Entertain* among a sample of Twitter users. Prior research has suggested that self-efficacy in information sharing, attachment motivation and critical mass explain retweeting motivations [20], which broadly contextualizes our findings in the realm of social cognitive theory. Similarly, findings predominantly align with Gruber (2017)’s findings wherein showing approval, arguing and gaining attention are predominantly interpersonal factors driving retweeting behavior.

Factors driving retweeting behaviors could be extrinsic (i.e., show approval, entertain) or intrinsic (i.e., argue, gain attention). Different motivations for retweeting could be instrumental in assessing or inferring reasons for user involvement in different topics or issues. This is especially so for specific demographic groups. Determining why people retweet could enable communication strategists to contextualize and gauge messages to the public online. Specifically, communication strategists could reach certain groups with targeted messages that elicit response (e.g., sending provocative messages to women who tend to engage/retweet through argument). Earlier research has suggested that retweeting is typically a measure of viral reach of information, such that the messages that receive the most retweets are considered to be the most influential [31]. This view, however, limits the understanding of this increasingly ubiquitous communication practice. The communicative meaning and valence of a tweet may change depending on what motivates the user to retweet and should be an area of future research. Additionally, while this study showed the reliability of the *Why We Retweet Scale*, it could not demonstrate its validity in relationship to prior reasons for retweeting. Future research should examine how the *Why We Retweet Scale* relates to existing measures of motivations to retweet including measures that include attention-seeking. Future research should also examine if these factors predict actual content of retweets.

Twitter recently introduced two changes that could make retweeting more powerful than before. For example, one change pertains to its algorithmic timeline, which exposes users to trending topics on top of their feed, which facilitates accelerated diffusion of popular tweets[32]. The other change includes a thread feature which allows users to string together tweets to serialize information [33]. Retweet references to these threads have the potential to engage a large audience with a longer story or thought or offer an in-depth commentary on an event or topic. These new features create opportunities for in-depth discussions about emerging topics. As a result, Twitter is likely to evolve as a communicative platform that encourages more nuanced exchanges. Coupled with the present study’s findings, it is critical to examine the underlying motivations for sharing information related to health, natural disasters, public policies, and governance.

## Limitations

This sample comprises Twitter users with public profiles limiting generalizability to those with private accounts. The sampling strategy (network clustering based on users’ tobacco-related terms) and sample size of this study also limits findings’ generalizability but are improvements over previous work [18]. The reliability of the *Argue* factor is lower than desired (Cronbach’s alpha = 0.6) and may be due to the number of items [34]. The convergent validity of this factor was also lower than desired (AVE = 0.42), however the Spearman-Brown coefficient approached an acceptable level. Replication, invariance testing (e.g., temporal, cultural), as well as other ongoing construct validity evaluation need to be considered in future research to better understand retweeting motivations.

## Conclusion

By developing the *Why We Retweet Scale*, this study provides a number of exploratory insights into the practice of online information dissemination. Instead of using counts of retweets as a reference to tweet virality or user engagement, this scale points to the user context, which lends meaningful interpretation of messages. For example, a policy decision maker would benefit from knowing whether the general public is retweeting about a proposed policy to express support for the policy or pursue their goal of building a network of like-minded individuals. Taken together, this scale informs communication strategists about factors associated with information propagation when designing campaign messages in order to maximize message research and engagement on Twitter.

## Supporting information

S1 Table Survey questions related to the measures. (DOCX)
