# Supplementary material for: The Why We Retweet scale
Source: PLoS One. 2018 Oct 18;13(10):e0206076. doi: 10.1371/journal.pone.0206076 (PMC6193720; doi:10.1371/journal.pone.0206076)
Supplement: S1 Table — (DOCX) [file pone.0206076.s001.docx]

| **Survey** | | |
| --- | --- | --- |
| 1 | Please enter your age in years | ______________ |
| 2 | Do you currently live in the United States? | - Yes - No |
| If age <18 years | |  |
|  | Thank you for your participation. Unfortunately, you are under 18 years of age and cannot legally proceed to the consent form and the rest of the survey. We apologize for any inconvenience this may have caused you. |  |
| If living outside of United States - Yes | |  |
|  | Thank you for your participation. Unfortunately, you must currently live in the United States to take the survey. We apologize for any inconvenience this may have caused you. |  |
| Consent | |  |
|  | Section Header: Please read the following Information Sheet. Your consent is required to proceed with this survey.  INFORMATION SHEET STUDY TITLE: Diffusion of Marketing Messages about Tobacco Products through Social Media - Study 3  PRINCIPAL INVESTIGATOR: Jennifer B. Unger, Ph.D.  DEPARTMENT: Preventive Medicine  TELEPHONE NUMBER: 323-442-8234  We invite you to take part in a research study. Please take as much time as you need to read this information. You may want to discuss it with your family or friends. This research study is sponsored by the National Institutes of Health. They provide funding to cover the costs of conducting this research study.  WHY IS THIS STUDY BEING DONE? This study is about how people use social media to discuss and learn about tobacco and nicotine products. We would like to compare the opinions and behaviors of people who post about tobacco on Twitter or Instagram with those of people who post about other topics. We hope to learn what people say online about tobacco and nicotine products, and how these messages affect other social media users. You are invited as a possible participant because you have recently posted on Twitter or Instagram about tobacco or about another topic. About 1800 participants will take part in the study.  WHAT IS INVOLVED IN THE STUDY? If you agree to take part, you will receive a link to an online survey. The survey will ask about your habits and interests, including your use of social media sites such as Twitter and Instagram and your use of specific tobacco and nicotine products. Six months after you complete the first survey, you will be invited to take another survey to see whether your habits and opinions have changed or remained the same. Each survey will take about 30 minutes to complete. You can do the surveys on a computer or mobile device such as a smartphone or tablet. Your study participation will end after you complete the two surveys. descriptive 10/12/2017 TCORS Study - Project 1, Study 3 \| REDCap https://redcap.sc-ctsi.org/redcap_v6.14.2/Design/data_dictionary_codebook.php?pid=3455 3/132  WHAT ARE THE POSSIBLE RISKS AND DISCOMFORTS? Some of the questions in the survey may make you feel uneasy or embarrassed. You can choose to skip or stop answering any questions that make you uncomfortable. There is a small risk that people who are not connected with this study will learn your identity or your personal information. WILL YOUR INFORMATION BE KEPT PRIVATE? Your answers will be kept in a password-protected and encrypted file. Only the research staff will have access to your answers. We will keep your responses for this study confidential as far as permitted by law. However, if we are required to do so by law, we will disclose confidential information about you. The University of Southern California's Institutional Review Board (IRB) may review your responses. The IRB is a research review board that reviews and monitors research studies to protect the rights and welfare of research participants. We may publish the information from this study in journals or present it at meetings. If we do, we will not use your name.  WHAT ARE THE POSSIBLE BENEFITS OF TAKING PART IN THIS STUDY? You will not receive any direct benefit from taking part in this study. However, your participation in this study may help us learn how messages about tobacco and nicotine products travel from one person to another. WHAT OTHER OPTIONS ARE THERE? An alternative would be not to take part in this study.  ARE THERE ANY PAYMENTS TO YOU FOR TAKING PART IN THE STUDY? You will receive a $20 Amazon gift card for completing each survey. If you receive more than $600 per year for taking part in one or more research studies, you may be required to pay taxes on that money. This does not include any payments you receive to pay you back for expenses like parking fees. You may receive an Internal Revenue Service (IRS) Form 1099 if you receive more than $600 in one year for taking part in one or more research studies.  WHAT ARE YOUR RIGHTS AS A PARTICIPANT, AND WHAT WILL HAPPEN IF YOU DECIDE NOT TO PARTICIPATE? Your participation in this study is voluntary. You are not giving up any legal claims or rights. If you do decide to take part in this study, you are free to change your mind and stop being in the study at any time.  WHOM DO YOU CALL IF YOU HAVE QUESTIONS OR CONCERNS? 10/12/2017 TCORS Study - Project 1, Study 3 \| REDCap https://redcap.sc-ctsi.org/redcap_v6.14.2/Design/data_dictionary_codebook.php?pid=3455 4/132 You may contact Jennifer B. Unger, Ph.D. at 323- 442-8234 with any questions, concerns, or complaints about the research or your participation in this study. If you have questions, concerns, or complaints about the research and are unable to contact the research team, contact the Institutional Review Board (IRB) Office at 323-223-2340 between the hours of 8:00 AM and 4:00 PM, Monday to Friday. (Fax: 323-224-8389 or email at [irb@usc.edu](mailto:irb@usc.edu)).  If you have any questions about your rights as a research participant, or want to talk to someone independent of the research team, you may contact the Institutional Review Board Office at the numbers above or write to the Health Sciences Institutional Review Board at LAC+USC Medical Center, General Hospital Suite 4700, 1200 North State Street, Los Angeles, CA 90033. | |
| 3 | Please select Yes if you consent to the above conditions? If you choose to not consent to the above conditions, please select No. (The survey will terminate.) | - Yes No |
|  | | |
| 4 | Please enter your Twitter username | _____________ |
|  | Below are some reasons why people might retweet a message. How often do you retweet for these reasons? |  |
| 5 | To show that I saw the tweet | - Never - Sometimes - Often - Very Often |
| 6 | To make more people see the tweet | - Never - Sometimes - Often - Very Often |
| 7 | To Spread knowledge | - Never - Sometimes - Often - Very Often |
| 8 | To entertain | - Never - Sometimes - Often - Very Often |
| 9 | To share a funny joke | - Never - Sometimes - Often - Very Often |
| 10 | To make my own twitter feed look good | - Never - Sometimes - Often - Very Often |
| 11 | To add my thoughts to a tweet | - Never - Sometimes - Often - Very Often |
| 12 | To get my followers to join the discussion | - Never - Sometimes - Often - Very Often |
| 13 | To say that I agree with the tweet | - Never - Sometimes - Often - Very Often |
| 14 | To argue against a tweet that I disagree with | - Never - Sometimes - Often - Very Often |
| 15 | To introduce my followers to the tweeter | - Never - Sometimes - Often - Very Often |
| 16 | To show my support for the tweeter | - Never - Sometimes - Often - Very Often |
| 17 | To show my followers that I like the tweeter | - Never - Sometimes - Often - Very Often |
| 18 | To show my followers how I feel about an issue | - Never - Sometimes - Often - Very Often |
| 19 | To tell my followers about an event | - Never - Sometimes - Often - Very Often |
| 20 | To gain new followers | - Never - Sometimes - Often - Very Often |
| 21 | To get someone's attentions | - Never - Sometimes - Often - Very Often |
| 22 | To save tweets so I can find them again | - Never - Sometimes - Often - Very Often |
| 23 | Because I trust the tweeter | - Never - Sometimes - Often - Very Often |
|  | | |
| 24 | Are you | - Male - Female |
| 25 | What is your ethnicity? | - Hispanic or Latnio - Not Hispanic or Latino |
| 26 | What is your race | - White - Black or African American - American Indian or Alaska Native - Asian/Pacific Islander - Other |
| 27 | What is your highest level of education currently? | - Less than high school - Some high school, no diploma - GED - High school graduate--diploma - Some college but no degree - Associate degree-- occupational/vocational - Associate degree--academic program - Bachelor's degree (ex: BA, AB, BS) - Master's degree (ex: MA, MS, MEng, Med, MSW) - Professional school degree (ex: MD, DDS, DVM, JD) - Doctorate degree (ex: PhD, EdD) - Other |
| 28 | What is approximate annual average income? | - Less than $10,000 2 - $10,000 to $14,999 3 - $15,000 to $24,999 4 - $25,000 to $34,999 5 - $35,000 to $49,999 6 - $50,000 to $74,999 7 - $75,000 to $99,999 8 - $100,000 to $149,999 9 - $150,000 to $199,999 - 10 $200,000 or more |
| **Thank you** | | |
|  | Thank you for sharing your opinions and information in this TCORS Study.  As a thank you, please complete the contact information below so that we may send you the unrestricted gift card.  As a reminder, your information will be kept strictly confidential, and will never be shared with any third party. We will follow-up with another survey in three (3) months should you choose to participate. Please enter your email address (example: ohnSmith@example.com) Your gift card and follow up survey will be sent to this email address. | |
